# Tips and tricks for laparoscopic interval transabdominal cervical cerclage; a simplified technique

**DOI:** 10.4274/jtgga.galenos.2019.2019.0028

**Published:** 2019-11-28

**Authors:** Yavuz Emre Şükür, Ertan Sarıdoğan

**Affiliations:** 1 Department of Obstetrics and Gynecology, Ankara University School of Medicine, Ankara, Turkey; 2 Clinic of Obstetrics and Gynecology, University College London Hospitals, London, United Kingdom

**Keywords:** Cervical cerclage, interval, laparoscopy, technique

## Abstract

With the advance of laparoscopic surgery, several minimally invasive cervical cerclage techniques have been described and the outcomes of those have been promising. With this video article, we describe a simplified technique for laparoscopic interval transabdominal cervical cerclage. The suture material is a standard non-absorbable, braided polyester Mersilene tape, which is also used for transvaginal cerclage. The straightened needle is passed medial to the uterine vessels and lateral to the cervico-isthmic junction in anteroposterior direction on both sides, and pulled out above the uterosacral ligament. The knot is tied posteriorly, just above the uterosacral plate. The advantages of straightened needles are easy insertion into the abdominal cavity through the 5-mm ports, and more accurate direction of the suture in anteroposterior direction. In addition, posterior knots can be removed via colpotomy in the event of pregnancy failure in the second trimester, and this allows vaginal delivery.

## Introduction

The two main indications for trans-abdominal cervical cerclage are grossly damaged cervical tissue due to previous surgeries or absence of vaginal portion of cervix, and previously failed elective vaginal cerclage (1). With the advance of laparoscopic surgery, several minimally invasive techniques have been described and the outcomes of those have been promising (2,3,4). With this video article, we describe a simplified technique that might reduce the risk of complications such as uterine artery or lower urinary tract injuries (Video 1).

### Technique

**Figure 1:**
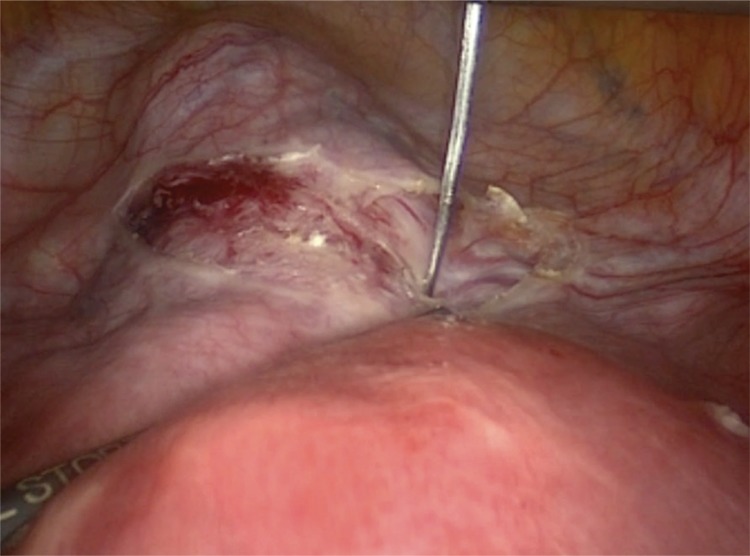
The needle is passed between the uterine vessels and cervico-isthmic junction with a right angle

**Figure 2:**
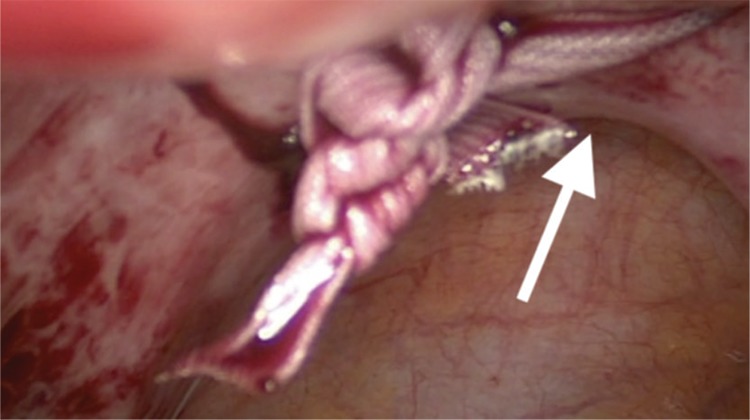
Knot-tied posteriorly, just above the uterosacral plate

**Figure 3:**
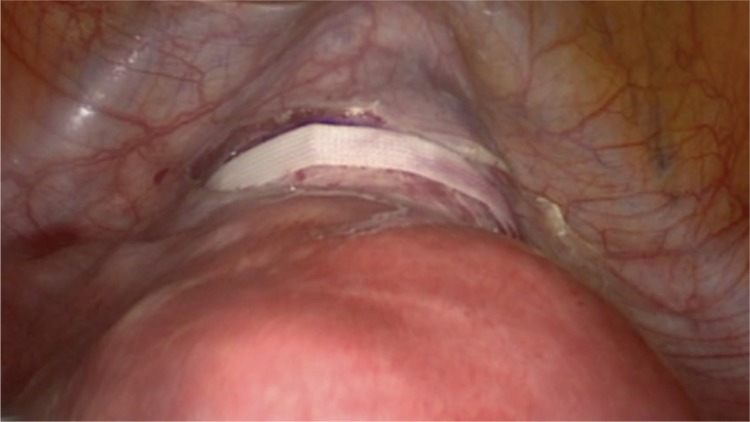
The tape is laid flat on the anterior surface of the uterus

Under general anaesthesia, the patient was positioned in a low dorsal lithotomy position in booted support stirrups. A urethral catheter was inserted prior to surgery. A uterine manipulated is placed into the endo-cervical canal to move the uterus during surgery and avoid obstruction of the cervical canal. The suture material is a standard non-absorbable, braided polyester Mersilene tape, which is also used for trans-vaginal cervical cerclage (Ethicon US, LCC, USA). First, the utero-vesical peritoneal fold is incised at the cervico-isthmic level and in order to identify the uterine vessels, the incision is extended laterally on both sides. Generally, the bladder is not reflected downwards. However, previous caesarean section or other anterior uterine surgeries that result in adhesions may necessitate dissection and bladder reflection. The straightened needle is passed medial to the uterine vessels and lateral to the cervico-isthmic junction in an anteroposterior direction with a right angle to the cervix (Figure 1), and pulled out from the posterior surface of broad ligament, 1 cm above the uterosacral ligament. Then, the same procedure is repeated on the left side. The knot is tied on the posterior surface of cervico-isthmic junction, just above the uterosacral plate (Figure 2). The Mersilene tape is carefully laid flat on the anterior surface of the cervix (Figure 3). The ends of the tape are cut at least 1 cm beyond the knot after tying. It is not essential to close the peritoneum on the anterior surface over the tape. After achieving haemostasis, the bladder catheter is removed if there is no contra-indication and the patient is discharged on postoperative day 0/1.

Although the suture may be inserted in either direction, we believe in that placing the suture from anterior to posterior has the advantages of better visualisation, and reduced risk of bowel injury and bladder erosions. In addition, posterior knots can be removed via colpotomy in the event of pregnancy failure in the second trimester and this allows vaginal delivery. Fibrosis can occur around and within the braided fibres of the Mersilene tape and make removal more difficult. However, a posterior knot can make it easy to remove when necessary.

The procedure can be simplified further by straightening the needles before insertion to the abdominal cavity. The two important advantages of straightened needles are easy insertion into the abdominal cavity through the 5 mm ports, and more accurate direction of the suture from the anterior to posterior direction at the cervico-isthmic level. An anterior knot may be beneficial to avoid adhesions in the Douglas pouch, and can also be easily removed in laparoscopy. However, it has the disadvantage of increased risk of bladder erosion.
